# Invited discussant comments during the UCL–Penn Global COVID Study webinar ‘Reflections, Resilience, and Recovery: A qualitative study of Covid-19’s impact on an international adult population’s mental health and priorities for support’: part 1 of 3

**DOI:** 10.14324/111.444/ucloe.100008

**Published:** 2022-12-01

**Authors:** Nigel Atter

**Affiliations:** 1 Policy Advisor, The British Psychological Society, UK

**Keywords:** psychological, people, support, Covid-19, older, old age, ageing, benefits, pensions, poverty, pandemic, loneliness, mental health, physical health, health, wellbeing

## Abstract

This discussant commentary will consider global health before the pandemic in relation to the UCL–Penn Global COVID Study survey results on what participants need to recover from the pandemic. It explores the case for expanding access to health care, the importance of culturally sensitive interventions and the need to scale up psychologically evidence-based interventions. Reflecting on the UCL–Penn Global COVID Study ‘Let’s Talk! What do you need to recover from Covid-19?’ webinar, the commentary highlights the recommendations from the British Psychological Society (BPS) to the government on what needs to happen for a better recovery.

**About the study**

The UCL–Penn Global COVID Study, launched, in April 2020, is a 12-month longitudinal study of the impact of Covid-19 on social trust, mental health and physical health. The Covid-19 global pandemic can be seen as a ‘natural stressor’ or major change in the environment that allowed researchers to study how changes in the environment can have an impact on individuals’ relationships with others and their health. In collaboration with six institutions from Italy, Singapore, the USA, China and the UK [1], the study looks at the short- and longer-term effects of Covid-19 on individuals’ mental health and social relationships with others. Survey data was collected at three timepoints: 17 April to 14 July 2020 (wave 1), 17 October 2020 to 31 January 2021 (wave 2) and 17 April to 31 July 2021 (wave 3).

**About the webinar**

Held online between 2 June and 28 July 2021, the study group presented study data at five online webinars as part of the UCL Global Engagement Fund sponsorship, to discuss the lessons learned. Policymakers and other subject experts were invited to speak on the policy relevance and implications of the study findings. A conscious decision was made by the study team to situate the study findings of individuals’ health and relationships in the context in which they occur – local communities and countries. The recorded comments from these discussions, focusing on the policy relevance and implications of each academic article, were recorded as discussant articles and published in this journal to be read alongside the research article being discussed.

These discussant articles are reviewed by members of the Editorial Board before being published. It is hoped that these discussant articles, read alongside the research articles, will provide a more holistic understanding of the issues at hand, how findings may inform policies in the coming months and/or assist in future crisis management strategies and aid decision making, in an open and transparent manner.

The study was pre-registered (https://osf.io/4nj3g/ on 17 May 2021) and ethical approval was obtained from the IOE (Institute of Education), UCL’s Faculty of Education and Society (University College London, UK) Ethics and Review Committee on 8 April 2020 (REC 1331) [2].

**Linked research article**

The linked research article to this discussion article cited here has been published in *UCL Open: Environment* following open peer review and made freely available to read as an open access article. Additionally, all previous versions and peer review reports are freely available to read as open access preprint articles from the journal’s preprint server by following the below DOI link and navigating to the version history of the published research article. Readers can find more information about how peer review works in the journal at ucl.scienceopen.com.

Wong KK, Loke K, Melville KMK. Reflections, resilience and recovery: a qualitative study of Covid-19’s impact on an international adult population’s mental health and priorities for support. *UCL Open: Environment*. 2022;(4):12. DOI: https://doi.org/10.14324/111.444/ucloe.000041.

**Recorded webinar**

This discussion article comments on the findings of the research article presented during the following webinar that has been recorded and made freely available to readers to watch on-demand.

Summer Webinar 5 – Let’s Talk! What do you need to recover from Covid-19? #GlobalCOVIDStudy. Available from: https://www.youtube.com/watch?v=i8z9KzIIcj0.

## Introduction

The UCL–Penn Global COVID study presented study findings on 28 July 2021. Participants were surveyed at three timepoints: 17 April to 14 July 2020 (wave 1), 17 October 2020 to 31 January 2021 (wave 2) and 17 April to 31 July 2021 (wave 3). The survey reached a global audience but most participants were from the UK. Across the three timepoints, it was found that mental health, physical health, work and personal relationships were stressful. Uncertainty about Covid-19, others not wearing masks and government guidelines had all become less stressful for participants by wave 3. Worries about future plans, boredom and loneliness and others not social distancing had remained about the same between waves. When asked what support was needed in the next six months, five key themes were identified:

1. Vaccines, freedom to travel and seeing people (40%)
2. Assurance from employers and financial support (23.4%)
3. Access to mental health services (19.6%)
4. Clarity of government guidelines (11.7%)
5. Understanding from others and personal support (5.3%).

However, the timeframe for recovery exceeded six months. Data from the survey supported three key topics to focus upon:

1. Relationships
2. Mental health and access to support
3. Bereavement and coping with loss.

Interventions and support needed to address relationship difficulties, mental health conditions, bereavement and loss will take time, commitment and additional funding.

## Discussant comments

Prior to the Covid-19 pandemic, significant progress was made in improving the lives, health and wellbeing of people across the globe. Life expectancy was increasing and mortality rates amongst children and mothers was improving [3].

However, depression and anxiety remained the most common mental health conditions, with 3–4% of the world’s population living with these afflictions at any given time. Together they are responsible, globally, for 8% of years lived with a disability [4].

Mental health conditions can emerge as a result of experiencing a range of psychosocial difficulties such as poverty or adverse childhood experiences (ACEs), which can increase the likelihood of experiencing anxiety and depression, interpersonal/domestic violence or drug and alcohol abuse, amongst other challenges.

### Expanding access to care

Psychologists know that prevention and early intervention are crucial to address premature mortality from non-communicable diseases and to promote mental health and wellbeing. Countries with the fewest mental health providers often have the most stressors, including violence, poverty, forced migration, social unrest or political instability. Between 75% and 85% of people with severe mental health conditions are unable to access the treatment they need in low- and middle-income countries, compared with 35% and 50% of people in high-income countries [5].

In the UK, a systematic review found that improving access to psychological therapy enabled large-scale access to effective evidence-based psychological therapies for large numbers of patients. The Improving Access to Psychological Therapies programme has transformed services for people with low-to-moderate anxiety and depression. It could potentially serve as a model for treatment in other countries [6].

### Cultural sensitivity

Psychologists and other health professionals have highlighted the importance of culturally appropriate approaches to psychosocial interventions [7]. Accessibility to such therapeutic provision is important, made more likely through ‘culturally sensitive’ care pathways and services [8,9]. In the UK, National Health Service (NHS) Trusts are working to provide inclusive, culturally sensitive mental health services. Psychologists play their part by ensuring that psychological assessments, formulations and interventions are written in accessible language which is culturally sensitive and non-discriminatory.

### Scaling up

There are many examples of good practice and psychologically informed interventions to alleviate poor mental health. The challenge is in scaling them up and ensuring they are accessible to community members. In 2013, the World Health Organization launched the first global mental health action plan, with a particular focus on how psychologists can train lay workers to deliver sophisticated psychological interventions to improve mental health, expanding the reach of psychology [10]. It is crucial that broad-based mental health policies encompass the impact of broader social contexts, such as poverty, racism, poor housing, violence and other stressors on individuals, families and communities.

All countries, whatever their wealth and income, can help find ways to address these issues. Action needs to be taken urgently to improve mental health and wellbeing through prevention, early intervention and treatment.

### UCL–Penn Global COVID Study – lessons from Let’s Talk! What do you need to recover from Covid-19?

The research presented during the webinar found that:

1. 24% of respondents wanted a reduction in workload – this would certainly improve wellbeing for many. Indeed, research supports this.
2. 24% of respondents wanted continued access to mental health services. The pandemic has had a significant impact on mental health across the population. NHS Digital, for example, has reported that mental health difficulties have increased from one in nine to one in six children. The sharp increase in eating disorders is particularly worrying.
3. 14% wanted greater understanding from others and more personal support. There have been excellent examples of communities pulling together to support those most in need.
4. Assurances from employers and financial support and help were highlighted by 12% of respondents. It is very concerning that, here in the UK, the £20 per week uplift in universal credit has been discontinued. Furthermore, disabled people have seen their benefits cut disproportionately. Additionally, research by the BBC [11] found that disabled needs have been forgotten during the pandemic. Thousands of disabled people spoke to the BBC to share their experiences during the crisis, with most saying their disability had become worse and over 2400 reporting that ‘routine, often vital, medical appointments had been cancelled’. A key worry was the reduction in access to care and support. The research found that 2604 people thought their mental health had got worse, and many signposted their instruction to shield at home as causing greater isolation and contributing to worse access to care. Scope, one of the UK’s largest disability charities, commented to say that the BBC’s findings ‘confirm the Government’s failure to provide support for disabled people throughout the pandemic’.
5. 11% of respondents wanted greater clarity from government guidelines and messaging. The Independent Scientific Advisory Group on which many eminent British psychologists sit have been advocating for exactly this.

#### So, what do we need to recover?

The BPS has lobbied the government to address:

- Multigenerational and ingrained poverty
- Educational attainment.

Kevan Collins, the former Government adviser on education recovery, plus international comparisons, has said ‘the recovery package is feeble in comparison with international programmes; this scale of shock requires a massive national effort to recovery, which is not just a bit of tutoring. Recovery should be the outcome of everything we do instead of having a narrow focus’.

In terms of funding wider wellbeing and mental health support in the UK, the Education Policy Institute stated that there is a gap in mental health support for children and young people. This also compares poorly internationally. The Education Policy Institute estimates the total funding in the UK is £310 per pupil, compared to £1800 in the US and £2100 per pupil in the Netherlands. In both of those countries, there is greater focus on wellbeing and on vulnerable groups.

The BPS has also lobbied the government to address:

- systemic racism
- inequality
- hunger and food insecurity
- insecure employment
- children and young people’s mental health
- neighbourhood violence
- lack of opportunity.

#### How should the UK Government address these issues?

- Developing a comprehensive, cross-departmental anti-poverty strategy.
- Ensuring that collaborative, multiagency working is the default approach at all levels of national, regional and local government – promoting and facilitating multiagency working to integrate health, education and social care services. These initiatives point toward the promise of population impact through psychological interventions that are delivered in a collaborative system of care.
- Services should be co-produced with people from the local community who will use them. It is essential to draw on individual and community expertise. It will empower and support local communities to transform economically disadvantaged areas.
